# Effect of Pioglitazone in Preventing In-Stent Restenosis after Percutaneous Coronary Intervention in Patients with Type 2 Diabetes: A Meta-Analysis

**DOI:** 10.1371/journal.pone.0155273

**Published:** 2016-05-10

**Authors:** Shi-jie Zhao, Zhao-shuang Zhong, Guo-xian Qi, Li-ye Shi, Ling Chen, Wen Tian

**Affiliations:** 1 Department of Geriatric Cardiology, First Affiliated Hospital, China Medical University, Shenyang, China; 2 Department of Respiratory, Central Hospital, Shenyang Medical College, Shenyang, China; Harvard Medical School, UNITED STATES

## Abstract

**Background:**

The benefits of pioglitazone in patients with type 2 diabetes mellitus (T2DM) after percutaneous coronary intervention (PCI) is unclear.

**Objectives:**

To evaluate the effect of pioglitazone on prevention of in-stent restenosis (ISR) in patients with T2DM after PCI.

**Methods:**

All full-text published relevant studies compared the effect of pioglitazone with control group (placebo or no pioglitazone treatment) on ISR in patients with T2DM after PCI were identified by searching the databases including PubMed, EMBASE, Cochrane Library and ISI Web of Science through October 2015. The endpoints were defined as the rate of ISR, late lumen loss, in-stent neointimal volume, target lesion revascularization (TLR) and major adverse cardiac events (MACE).

**Results:**

Six studies (5 RCTs and 1 retrospective study), comprising 503 patients, were included into this meta-analysis. In the pioglitazone group, as compared with the control group, the risk ratio for ISR was 0.48 (I^2^ = 14.5%, P = 0.322; 95%CI 0.35 to 0.68, P<0.001), the risk ratio for TLR was 0.58 (I^2^ = 6.0%, P = 0.363; 95%CI 0.38 to 0.87, P = 0.009). The result showed there was no association between the use of pioglitazone and the events of MACE (I^2^ = 36.7%, P = 0.209; RR 0.56, 95%CI 0.30 to 1.05, P = 0.071). For the considerable heterogeneity, further analysis was not suitable for the endpoints of late lumen loss (I^2^ = 81.9%, P<0.001) and neointimal volume (I^2^ = 75.9%, P = 0.016).

**Conclusions:**

The treatment of pioglitazone was associated with a reduction in ISR and TLR in T2DM patients suffering from PCI, except the incidence of MACE.

## Introduction

In-stent restenosis (ISR) is considered as the leading problem after percutaneous coronary intervention (PCI), especially in patients with type 2 diabetes mellitus (T2DM)[1,2]. Thiazolidinediones (TZDs), agonists of the peroxisome proliferation-activated receptor-γ (PPAR-γ), usually used to improve insulin sensitivity and reduce blood glucose levels in diabetic patients, could also inhibit proliferation and migration of vascular smooth muscle cells (VSMCs) and ameliorate inflammation after vascular injury[3–5]. Among the three TZDs, pioglitazone may be the only one which shows beneficial effects on cardiovascular outcomes and appears to be promising in clinical application[6–8]. Previous meta-analysis has suggested that pioglitazone is effective in decreasing ISR and the need for revascularization after bare-metal stents (BMS) implantation in diabetic patients[9]. However, drug-eluting stents (DES) are more widely used in recent years. Diabetes mellitus was also regarded as one of the most powerful clinical predictors of ISR after DES implantation as same as in BMS era[10–12]. A hypothesized independent anti-restenotic effect of pioglitazone in patients with T2DM has not been clearly demonstrated[13]. Thus, we performed this meta-analysis to investigate the effect of pioglitazone on prevention of ISR in patients with T2DM after PCI, with update information both on BMS and DES implantation.

## Methods

This meta-analysis was conducted according to the PRISMA statement [14], and the PRISMA checklist is provided as S1 Table.

### Search Strategy

We searched the databases including PubMed (http://www.ncbi.nlm.nih.gov/pubmed), EMBASE (http://www.embase.com), Cochrane Library (www.cochranelibrary.com) and ISI Web of Science (http://www.webofknowledge.com) for all full-text published relevant studies compared the effect of pioglitazone with control group (placebo or no pioglitazone treatment) in T2DM patients after PCI through October 2015. Search terms included any possible combination of the keywords of ‘‘restenosis”, ‘‘percutaneous coronary intervention or PCI”, ‘‘pioglitazone” and ‘‘diabetes”.

### Study selection

Two independent reviewers (S.-J.Z. and L.C.) were involved in the search for potentially eligible studies and in the inclusion process. In case of disagreement, a decision was made under the supervision of the senior author (W.T.). Studies will be included if they met the following criteria: (1) RCT or high quality retrospective study, (2) pioglitazone was administered and compared with control group (placebo or no pioglitazone treatment) for patients who suffered from diabetes and performed PCI, and (3) the studies included at least one of the following interesting outcomes: ISR (defined as stenosis more than 50% at the site of stent), late loss (change in minimum lumen diameter at the stent site from baseline to follow-up), in-stent neointimal volume (neointimal volume/length of stent) measured by intravenous ultrasound (IVUS), target lesion revascularization (TLR) and major adverse cardiac events (MACE, including target vessel revascularization, acute myocardial infarction, cardiac death, etc.).

### Data abstraction and quality assessment

For all included studies, we recorded the baseline information of the study, including the number of subjects, patients’ characteristics, interventions in each group, methodology quality of the study, outcomes with ascertained method and length of follow-up. The data were extracted independently by two authors(S.-J.Z. and L.C.) using a pre-designed data extraction form. As before, disagreements will be resolved by discussion and referral to the senior author(W.T.) if necessary.

### Statistical analysis

The treatment effect for continuous outcomes are expressed as weighted mean difference (WMD) with 95% confidence intervals (CI) for all studies used the same scale[15]. For dichotomous data, the pooled risk ratios (RRs) would be used. Statistical heterogeneity across the studies was measured by I^2^ test, and a fixed-effects (FE) model (Mantel-Haenszel method) would be used if I^2^≤50%. In case of important heterogeneity (50%<I^2^≤75%), further exploration was made if appropriate, including sensitivity analysis and subgroup analysis. If heterogeneity remained significant, a random-effects (RE) model would be used. If there was considerable heterogeneity (I^2^>75%), the data would be regarded as unsuitable for pooling[15]. The publication bias was evaluated by using funnel plots with Begg’s test[16]. A value of P<0.05 indicated statistically significant. All analysis was completed in Stata v12.0 (Stata Corp, College Station, TX,USA) using the metan function.

## Results

### Baseline characteristics of included studies

Our search strategy resulted in the initial identification of 125 records. After duplicated removal, 91 studies were left, in which 81 records were further excluded as non-clinical studies, reviews, case reports or irrelevant studies after the title and abstract screening. As a result, a total of 10 studies were left for full-text screening to determine eligibility for analysis.

**Fig 1:**
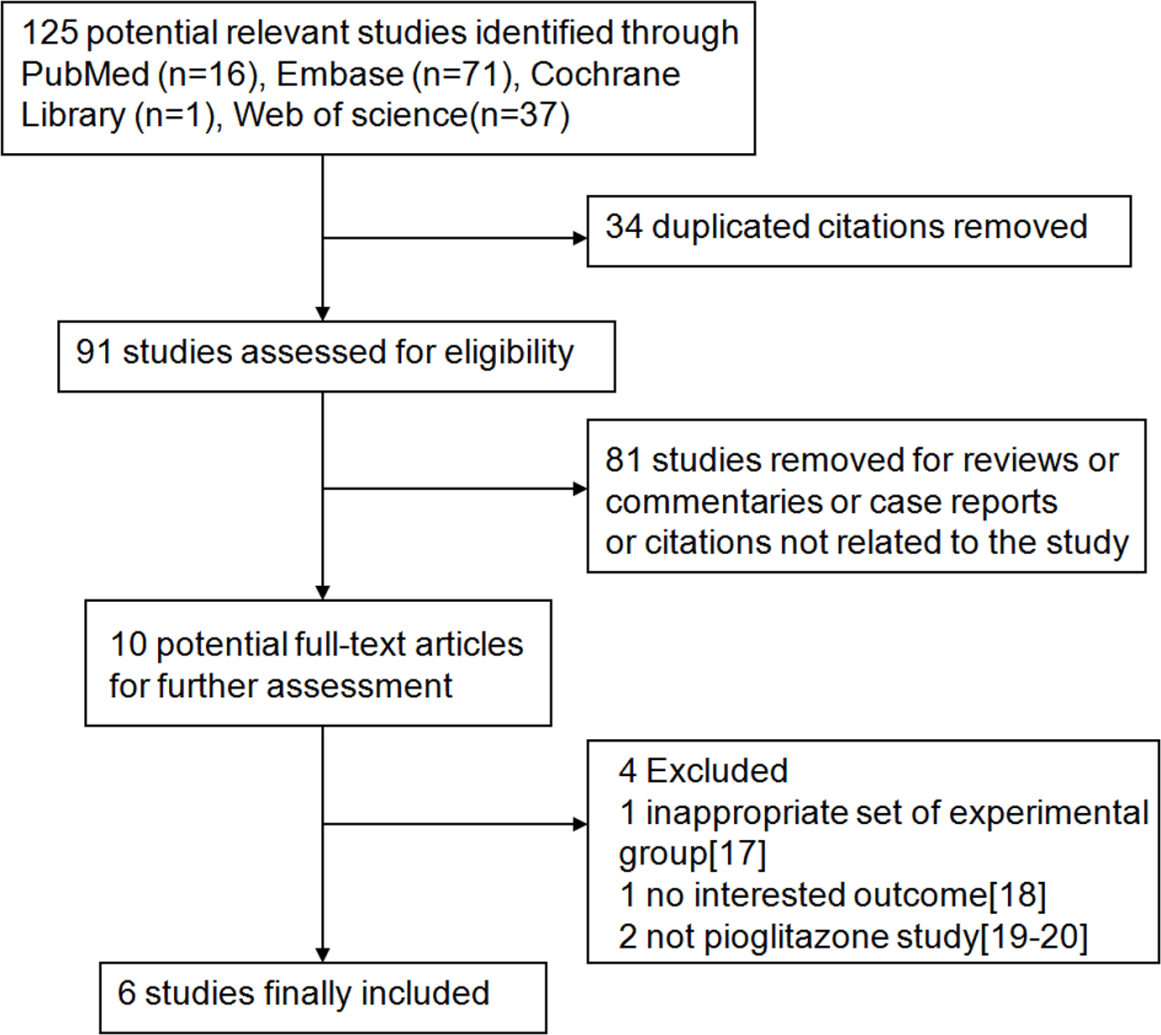
Flow chart of study selection.

After full-text screening, 4 studies were removed due to either inappropriate set of experimental group, lack of interested outcome or other reasons[17–20]. Finally, 6 studies (5 RCTs and 1 retrospective study) involved a total of 503 cases, were selected for inclusion in the meta-analysis. A flow chart summarizes the selection process of the studies (Fig 1), and the detail information of the included studies is provided in Table 1[21–26].

**Table 1:** Baseline characteristics of selected studies.

Study	Year	Study design	Age(PG/CG)	Sample size(PG/CG)	Stent type	Interventions		Outcomes		Follow-up
						PG	CG	Routine Follow-up	Clinically Driven	
Lee, H et al.[21]	2013	RCT	60±10/62±9	60/61	DES	pioglitazone, 15 mg/d	placebo	ISR; Late loss; Neointimal volume; MACE	TLR	12 months
Soon Jun Hong et al.[22]	2011	RCT	64±7/62±8	47/47	DES	pioglitazone, 30 mg/d	placebo	ISR; Late loss; Neointimal volume; MACE	-	8 months
Takagi, T et al.[23]	2009	RCT	64±9/62±10	48/49	BES	pioglitazone, 30 mg/d	placebo	ISR; Late loss; MACE	TLR	6 months
Yokoyama, J et al.[24]	2007	Retrospective	63±10/64±9	56/37	BES	pioglitazone, 15 mg/d	without pioglitazone	ISR; Late loss	TLR	6 months
Nishio, K et al.[25]	2006	RCT	66±9/68±10	26/28	BES	pioglitazone, 30 mg/d	without pioglitazone	ISR; Late loss	-	6 months
Takagi, T et al.[26]	2003	RCT	64±10/65±9	23/21	BES	pioglitazone, 30 mg/d	without pioglitazone	ISR; Neointimal volume	TLR	6 months

PG = pioglitazone group; CG = control group; DES = drug-eluting stent; BES = bare-metal stent; ISR = in-stent restenosis; TLR = target lesion revascularization; MACE = major adverse cardiac events.

### The quality assessment and publication bias

**Fig 2:**
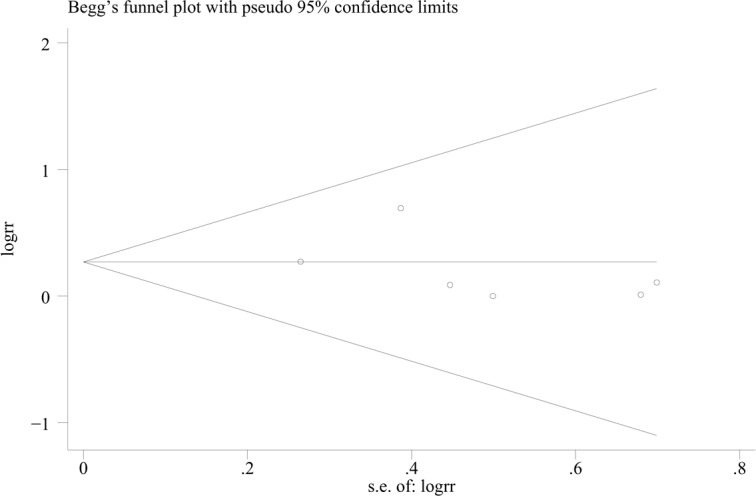
Funnel plot for the events of ISR.

The assessment of study quality was performed by application of Modified Jadad scale, which is composed of randomization method, double-blinding, withdrawals and dropouts and allocation concealment[27]. The Jadad scores of the studies are summarized in Table 2. The publication bias was assessed by a funnel plot, which was based on the outcome of ISR (Fig 2), and no publication bias was found (Begg’s test, P = 0.707).

**Table 2:** Methodologic quality assessment of selected studies.

Author	Randomization	Randomization method	Blinding	Withdrawals/dropouts	Allocation concealment	Scores
Lee, H et al.[21]	Yes	Unclear	Unclear	No	Unclear	3
Soon Jun Hong et al.[22]	Yes	Unclear	single blind	Yes	Unclear	3
Takagi, T et al.[23]	Yes	Unclear	open label	No	Unclear	2
Yokoyama, J et al.[24]	No	No	No	No	No	0
Nishio, K et al.[25]	Yes	Unclear	Unclear	No	Unclear	3
Takagi, T et al.[26]	Yes	Unclear	double blind	No	Unclear	4

### Statistical Analysis Results

#### In Stent Restenosis

**Fig 3:**
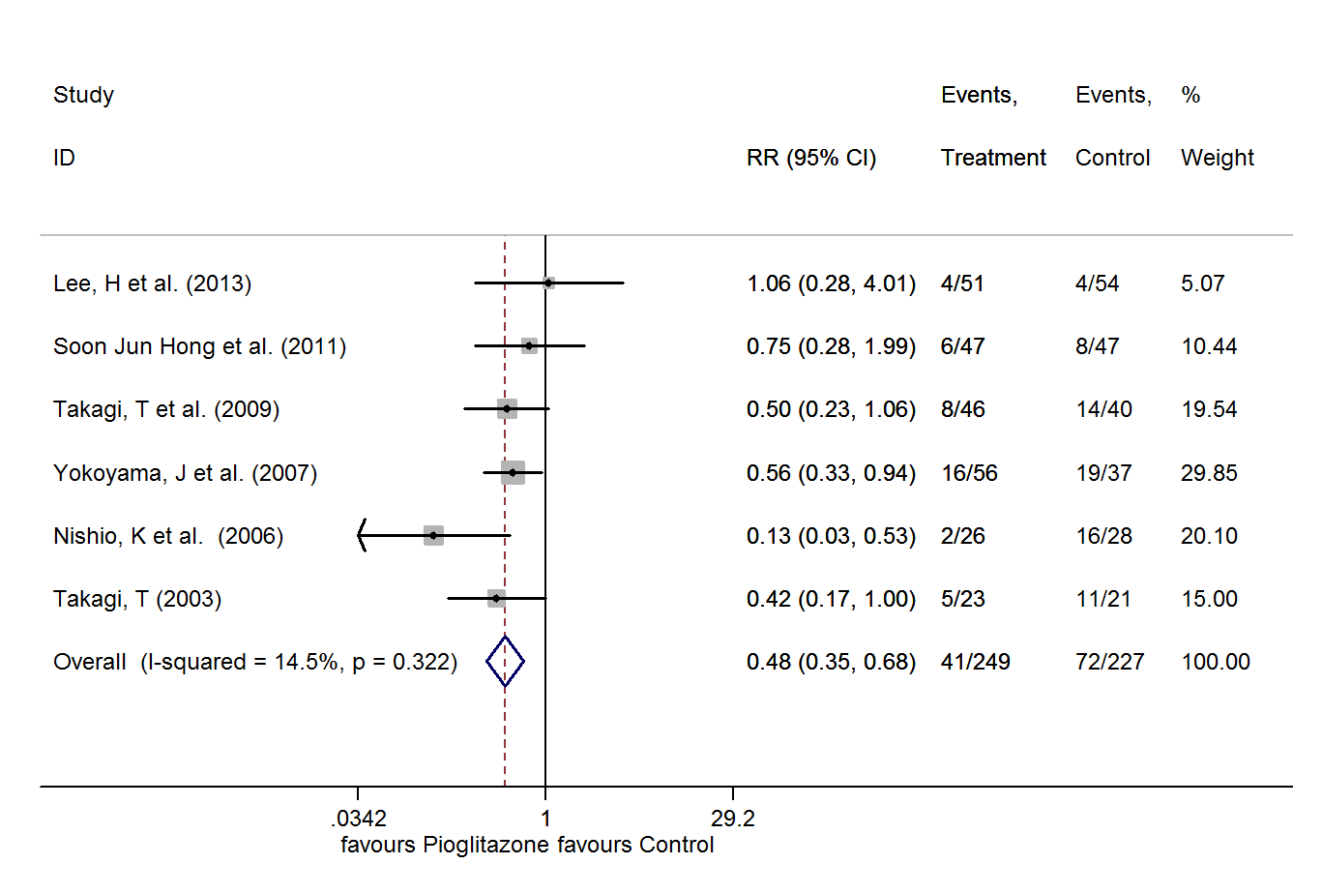
RR of the events of ISR.

There was no significant heterogeneity among the six studies[21–26] (I^2^ = 14.5%, P = 0.322), so the FE model was used. In the six studies, the events of ISR occurred in 41 of 249 patients (16.5%) treated with pioglitazone and in 72 of 227 patients (31.7%) without pioglitazone treatment. The use of pioglitazone was associated with a significant reduction in the events of ISR (RR 0.48, 95% CI 0.35 to 0.68, P<0.001) (Fig 3).

#### Late Loss

The late loss was reported after pioglitazone treatment in the diabetic patients suffered from PCI in 5 studies[21–25]. There was considerable heterogeneity among these studies (I^2^ = 81.9%, P<0.001). As a result, the data about late loss in the studies were not suitable for further analysis.

#### Neointimal Volume

The neointimal volume of the target lesion was investigated by IVUS at 1 year follow-up in 3 studies[21,22,26]. After pooling the data of the studies, we were not able to perform further analysis for the results of neointimal volume because of the considerable heterogeneity among the studies (I^2^ = 75.9%, P = 0.016).

#### Target Lesion Revascularization

**Fig 4:**
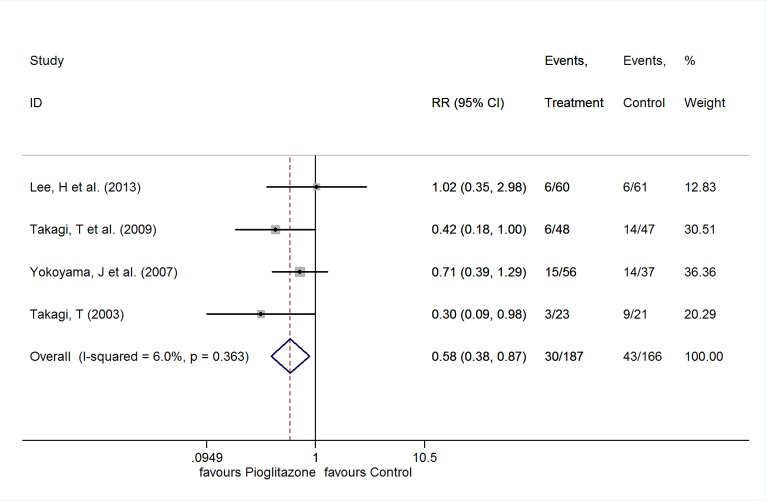
RR of the events of TLR.

The meta-analysis was performed with FE model for there was no significant heterogeneity among the 4 studies [21,23,24,26] (I^2^ = 6.0%, P = 0.363). Pioglitazone treatment was associated with a significant reduction in the events of TLR (RR 0.58, 95%CI 0.38 to 0.87, P = 0.009) (Fig 4).

#### Major Adverse Cardiac Events

**Fig 5:**
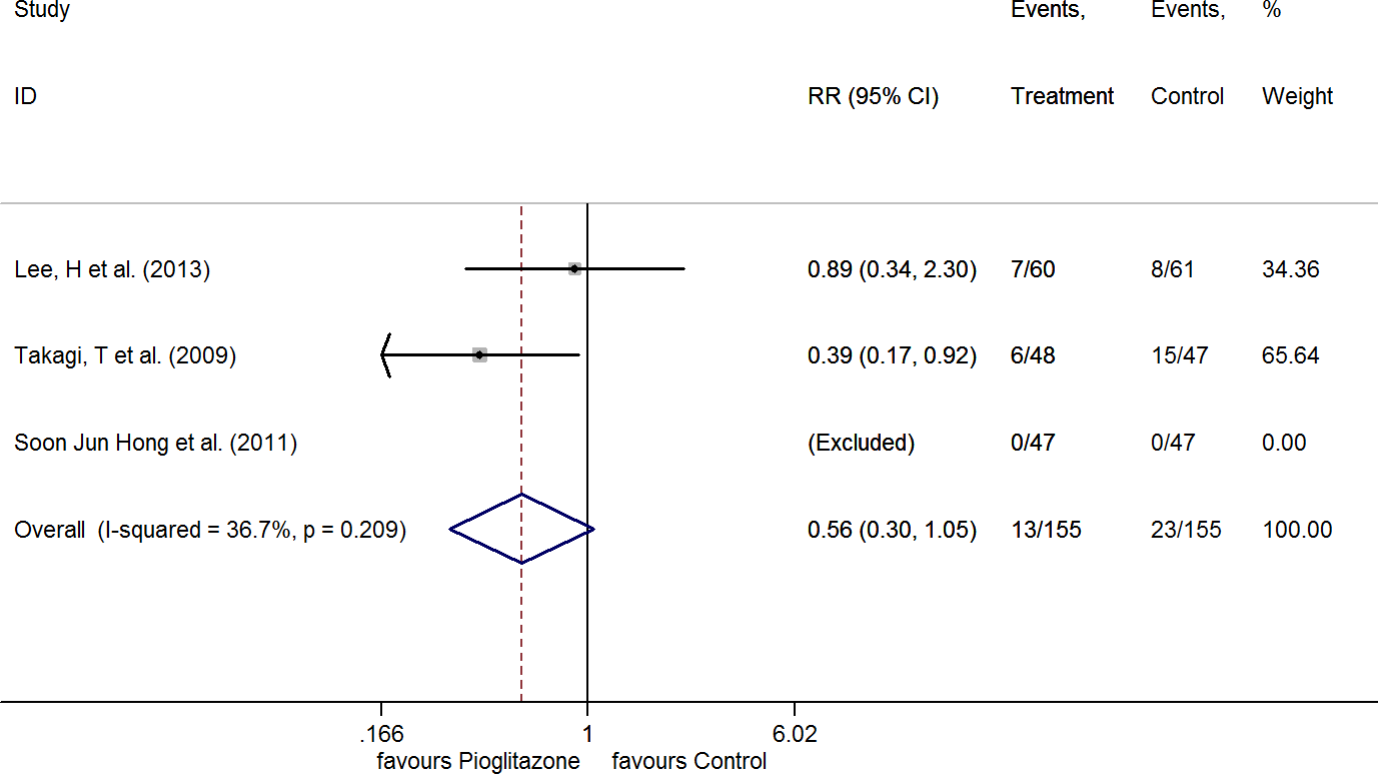
RR of the events of MACE.

The incidence of MACE was reported in three studies[21–23], and there was no significant heterogeneity among them (I^2^ = 36.7%, P = 0.209). After the application of FE model for the meta-analysis, the result showed there was no association between the treatment of pioglitazone and MACE (RR 0.56, 95%CI 0.30 to 1.05, P = 0.071) (Fig 5).

## Discussion

Inflammation response evoked by stent implantation, which initiates several vascular remodeling processes including neointimal hyperplasia, is crucial contributor to the development of restenosis after PCI[28], especially in patients with T2DM[29]. As anti-diabetic agents involved in insulin sensitivity and lipid metabolism, TZDs can activate PPAR-γ, which is highly expressed in activated macrophages after PCI[30], to exert its anti-inflammatory and anti-proliferative effect in the process of ISR[31–34]. However, among the three TZDs, troglitazone is not approved due to its liver toxicity[8], and rosiglitazone is associated with increased risk of myocardial infarction[6]. So, as the only one which shows beneficial effects on cardiovascular outcomes in TZDs, pioglitazone is considered to be a more promising drug on the prevention of ISR[23,35].

Although the incidence of ISR was markedly reduced since DES have been widely used [36,37], a fairly high rate of ISR (10.1% to 17.6%) after DES implantation still exists in patients with T2DM[10,38], which probably due to the amplified inflammatory response and increased neointimal hyperplasia. Therefore, diabetes is one of the most powerful clinical predictors of ISR after PCI regardless of the kind of stents used[1,10,11,39]. Besides, little data is available about the dosage of pioglitazone that can appropriately prevent ISR after PCI with DES, especially in T2DM patients. Therefore, we made the meta-analysis without selection of the kind of stent and the dosage of pioglitazone, which might mainly account for the considerable heterogeneity of late loss and neointimal volume. Nevertheless, as the results have shown, no statistical heterogeneity was found among the included studies for most endpoints, indicating that the results of the study are convincing in some degree.

This study, comprising 503 T2DM patients, demonstrates that the use of pioglitazone was associated with a reduction in ISR and TLR. Although late loss and neointimal volume were not further analyzed due to the considerable heterogeneity among the studies, the beneficial effects of pioglitazone on these endpoints could not be ignored [22,23,25,26]. The benefits of pioglitazone for T2DM patients could be attributed to its favorable effect on lipid profile[40], attenuation of atherosclerotic plaque inflammation[4] and efflux of cholesterol from macrophages[41]. In this regards, Pioglitazone could be an useful agent on the prevention of ISR [42–44]. However, it is worth noting that no studies, including this meta-analysis, have found that pioglitazone reduces cardiovascular events, even the best available data for pioglitazone from the PROactive trial[45]. For this reason, the results of the study should be interpreted with care and more high quality clinical trials are needed.

Several limitations should be considered in this study. First, for only six studies met our inclusion criteria, the publication bias of the study could not be totally excluded for the low power of funnel plot asymmetry test. Second, the considerable heterogeneity mentioned above, together with the small sample size, lower quality of the RCT trials (Modified Jadad score ranged from 0–4) and the enrollment of one retrospective study, could make the conclusion less convincing. Finally, the lack of subgroup analysis, such as condition of the patients, selection of the stents and the dosage of pioglitazone might also lead to significant bias in the study. Therefore, the results of the study should be interpreted with caution and additional clinical trials are required.

## Conclusions

The limited evidence indicates that the treatment of pioglitazone is associated with a reduction in ISR and TLR in T2DM patients suffering from PCI, except the incidence of MACE. However, due to the limitations of the present study, additional high quality RCTs are needed and the results of the study should be interpreted with care.

## Supporting Information

S1 Table PRISMA Checklist. (DOC)
